# Inflammatory Process on Knee Osteoarthritis in Cyclists

**DOI:** 10.3390/jcm12113703

**Published:** 2023-05-26

**Authors:** David Noriega-González, Alberto Caballero-García, Enrique Roche, Melchor Álvarez-Mon, Alfredo Córdova

**Affiliations:** 1 Department of Surgery, Ophthalmology, Otorhinolaryngology and Physiotherapy, Faculty of Medicine, HVUV, 47003 Valladolid, Spain; 2 Department of Anatomy and Radiology, Faculty of Health Sciences, GIR Physical Exercise and Aging, University of Valladolid, Campus Los Pajaritos, 42004 Soria, Spain; 3 Department of Applied Biology-Nutrition and Institute of Bioengineering, Miguel Hernández University (UMH), 03202 Elche, Spain; 4 Institute for Health and Biomedical Research (ISABIAL), 03010 Alicante, Spain; 5 CIBER Fisiopatología de la Obesidad y la Nutrición (CIBEROBN), Instituto de Salud Carlos III (ISCIII), 28029 Madrid, Spain; 6 Department of Internal Medicine, University of Alcalá de Henares, 28801 Alcalá de Henares, Spain; 7 Department of Biochemistry, Molecular Biology and Physiology, Faculty of Health Sciences, GIR Physical Exercise and Aging, University of Valladolid, Campus Duques de Soria, 42004 Soria, Spain

**Keywords:** cartilage, chondrocytes, collagen, cytokines, joint, pedaling

## Abstract

Osteoarthritis is a disorder affecting the joints and is characterized by cellular stress and degradation of the extracellular matrix cartilage. It begins with the presence of micro- and macro-lesions that fail to repair properly, which can be initiated by multiple factors: genetic, developmental, metabolic, and traumatic. In the case of the knee, osteoarthritis affects the tissues of the diarthrodial joint, manifested by morphological, biochemical, and biomechanical modifications of the cells and the extracellular matrix. All this leads to remodeling, fissuring, ulceration, and loss of articular cartilage, as well as sclerosis of the subchondral bone with the production of osteophytes and subchondral cysts. The symptomatology appears at different time points and is accompanied by pain, deformation, disability, and varying degrees of local inflammation. Repetitive concentric movements, such as while cycling, can produce the microtrauma that leads to osteoarthritis. Aggravation of the gradual lesion in the cartilage matrix can evolve to an irreversible injury. The objective of the present review is to explain the evolution of knee osteoarthritis in cyclists, to show the scarce research performed in this particular field and extract recommendations to propose future therapeutic strategies.

## 1. Introduction

The Osteoarthritis Research Society International (https://www.google.com/search?client=firefox-b-d&q=OARSI, accessed on 24 March 2023) has defined osteoarthritis (OA) as a disorder affecting mobile joints, presenting cell stress and cartilage degradation of the extracellular matrix (ECM). The inadequate repair of micro- and macro-lesions can trigger this degenerative process, activating a proinflammatory response. In 1995, the World Health Organization (WHO) defined OA as “the result of mechanical and biochemical stress that destabilize the balance between the synthesis and degradation of cartilage and subchondral bone”. This imbalance seems to be multifactorial, where genetic, developmental, metabolic, and traumatic factors may be responsible.

OA affects all tissues of the joint. At the cellular level, chondrocytes are directly affected. These cells are responsible for the remodeling (synthesis-degradation) of ECM [1], evidenced by molecular, morphological, and functional-biomechanical changes. This in turn leads to fissures and ulcers in articular cartilage and can affect the subchondral bone (osteophytes and cysts). Pain, joint deformation, disability, and local inflammation are the main symptoms of OA.

In sports, the appearance of OA also depends on particular factors, such as the type of exercise and the biomechanical intensity of performance. The present review will focus on cycling. This sport discipline has no weightlifting and requires negligible eccentric contractions. Unlike running, football or any other aerobic exercises whose performance involves repeated eccentric contractions [2,3], cycling involves repeated concentric contractions, conditioned by adjustments in the height of the saddle at the time of maximum internal abduction of the knee. This is an important load variable for the medial compartment of the tibiofemoral joint. In this sense, it has been observed that the increase in the moment of knee extension is related to the decrease in the height of the saddle, which implies an increase in the load of the knee joint. In other eccentric sports such as football or basketball, players perform characteristic movements, such as repeated jumps and sprints, requiring great effort from joints and muscles that can result in muscle damage [2,4]. In these disciplines, the knee joint also undergoes an inflammatory process, whose severity depends on the intensity and duration of the training session or competition. In this sense, high-intensity training can increase basal cortisol levels, which may in turn cause inflammation in the joints [5].

The pain caused by knee OA is more intense during a period of movement, and decreases when resting. However, in the most severe cases of knee OA, pain occurs even at rest [6]. In both the early and late stages, synovial inflammation usually occurs in most patients with OA. In this sense, macrophages play a key role in the pathogenesis of OA through the production of inflammatory mediators, growth factors and proteinases [7]. At the moment, OA is a disease with no stablished therapy and is characterized by the progressive deterioration of articular cartilage associated with subchondral and osteophytic bone proliferation, which causes pain, limited mobility, disability and deterioration of the patient’s quality of life [8]. Thus, OA cannot be described as a single disease, but as a heterogeneous group of disorders with similar clinical manifestations and common pathological and radiological changes [9].

The knee does not undergo major impacts in cycling. However, the continuous concentric movement (sustained pedaling motion with intensity changes) produces microtrauma. Pedaling biomechanics is complex, since the natural movement of the knee is conditioned by the constant fixation point of the forefoot. In this context, forefoot fixation is necessary for optimal performance during pedaling, but at the same time, pedaling repetition is the cause of the aforementioned microtraumas. Approximately 15,000 to 20,000 pedal strokes occur over 100 km. This movement is not a flexo-extension of the knee caused by contractions of the quadriceps muscle, but rather involves the iliac hamstring and psoas muscles. In addition, the knee works with a controlled rotation of the tibia over the femur due to the external rotator muscles [10]. Therefore, the aim of this review is to explain knee OA in more detail and to present the few works that have studied knee OA in cyclists and the treatments performed. The next step is to implement future research, trying to reduce the incidence of joint injuries in the long term.

## 2. Etiopathogenesis of Knee Osteoarthritis: Risk Factors

Joint cartilage displays low regenerative capacity due to the absence of blood vessels and neuronal connections, as well as the postmitotic phenotype of adult chondrocytes. This tissue lines the surface of the synovial joints, providing a frictionless sliding surface for shock absorption during locomotion [11]. The high water content of the ECM and the specific orientation of collagen fibers are in part responsible for the carrying capacity and flexibility of articular cartilage [11].

As previously mentioned, OA presents a complex multifactorial etiology [12,13]. Risk factors for the development of knee OA can be due to endogenous (genetics, age, sex, obesity) or exogenous (other injuries, inadequate joint alignment, excessive loading, instability) factors [14,15,16,17]. In this context, strenuous physical activity, especially activities requiring kneeling, knee flexion, squats, and prolonged standing, along with trauma and injury have been linked to a high prevalence of knee OA [18]. However, these actions are not frequent in cycling. In this sport discipline, all conditions associated with knee OA are mainly due to overuse of the joint. This can lead to axial deviations (especially genu, varus or valgus), meniscopathy, or intra-articular ligament injuries, which would aggravate the arthritic situation during movement. However, due to the multifactorial nature of OA, other poorly characterized factors may still need additional research.

There has been recent interest in relation to the genetic factors that may increase the risk of developing OA. In this sense, more than 80 genes related to this pathology have been identified. Some of these encode vitamin D receptors or insulin-like growth factors (IGF-1) [19]. In addition, there are studies that confirm a 40% heritability for knee OA. However, these genes are not unique to this pathology, being also present in other pathological processes. Nevertheless, a key gene associated with the development of OA is Growth Differentiation Factor-5 (GDF-5), characterized in studies on Turkish populations [20].

On the other hand, confounding factors should also be considered. Patellar chondromalacia and inflammation of the thigh muscles can evolve to OA. Patellar chondromalacia is the most frequent pathology, and is considered quite painful. Histologically, a lesion circumscribed to the patellar cartilage is observed as a premature degeneration of this tissue. This alteration can appear along with edema, fibrillation, fissures, and even rupture, depending on the degree of the injury. Therefore, patellar chondromalacia is considered as a pre-arthritis condition for the knee [21]. On the other hand, inflammation spreading from thigh muscles can contribute to the development of knee OA. In this context, cyclists may have stronger quadriceps, which protects (around 30%) against knee OA progression [22].

## 3. The Inflammatory Process in Knee Osteoarthritis

Knee OA has an inflammatory component due to the presence of proinflammatory cytokines, reactive oxygen species (ROS), nitric oxide (NO), degrading enzymes of ECM, and biomechanical stress. As mentioned previously, cyclists suffer permanent microtraumas due to the effort required for continuous pedaling. In this context, the chondrocytes present altered catabolic and anabolic activities, resulting in an unbalanced turnover of the ECM in the articular cartilage. This results in accelerated destruction of ECM, followed by deficient cartilage repair [23]. Altogether, the synthesis of proteins of the surface area of cartilage (i.e., lubricin) is altered, evolving to a suboptimal surface lubrication and thereby an increase in friction and cartilage deterioration [24,25,26].

Proteoglycan content of cartilage surface can also be reduced, and certain parts of the type II collagen network are eroded and exposed. Superficial chondrocytes change the spatial organization and form clusters. Small fragments of collagen are released, activating an inflammatory cascade within the cartilage, extending inflammation within the synovial membrane and generating a stroke [27,28]. This catabolic situation causes changes in the composition of ECM, resulting in a decrease in the water-fixing capacity that leads to further deformation of the cartilage when subjected to loading actions [29,30].

The synovial inflammation mentioned may appear in early and late stages in patients with OA. Therefore, high levels of proinflammatory cytokines have been detected in the synovial zone of affected individuals [31]. In addition, macrophages play an instrumental role in the pathogenesis of OA, as they stimulate inflammation and produce growth factors and proteinases [32]. In this context, the overproduction of cytokines and growth factors in the inflamed synovial membrane stimulates the expression of matrix metalloproteinases (MMPs), favoring ECM degradation [7]. In addition, chondrocytes produce cytokines and other inflammatory mediators, such as IL (interleukin)-1β, IL-1Ra, IL-6, IL-8, IL-12, IL-17, IL-18, TNF-α, and prostaglandins (PG) such as PGE2 [33,34]. Chondrocytes stimulated by proinflammatory messengers such as IL-1β produce NO, which would inhibit proliferation and stimulate apoptosis in this cell type [35,36]. NO is produced by inducible NO synthase (iNOS). NO favors the expression of TNFα and IL-1β and the activation of MMPs. At the same time, NO inhibits the expression of IL-Ra (IL-1 receptor antagonist), which blocks the action of IL-1β [37,38,39]. Along with the overproduction of NO, alterations in the mitochondrial respiratory chain of arthritic chondrocytes could also contribute to the apoptosis of this cell type through the uncontrolled production of ROS [40].

Growth factors, such as IGF or TGF-β (transforming growth factor-β), are not capable of counteracting the destructive effect of the proinflammatory mediators mentioned. Therefore, an imbalance of the pro-/anti-inflammatory system appears in pathological conditions, resulting in the activation of proteolytic enzymes, such as the aforementioned MMPs and ADAMTs (A disintegrin-like and metalloproteases with thrombospondin), destroying the ECM of cartilage and altering tissue architecture [41,42,43].

Finally, it is unknown whether in addition to chondrocytes and macrophages, there are other cell types that generate proinflammatory mediators, such as cells from other intra-articular tissues [36,44,45]. In this context, it has been described that some of the most active proinflammatory mediators in the pathogenesis of OA (IL-1β, TNF-α and IL-6) favor the inflammatory process by stimulating the production of other inflammatory cytokines by other cell types, contributing to disease progression [46]. This inflammatory situation defines OA as a specific degenerative disease, most likely requiring specific regenerative treatments for remission [47]. Nevertheless, more research is necessary to study more specifically the inflammatory process on knee osteoarthritis in cyclists.

## 4. Knee Osteoarthritis in Cycling

Mature joint cartilage is composed of a specific ECM with type II collagen fibers in a particular orientation. Such fibers are partly responsible for the load capacity and flexibility of the joint. In the superficial layer of joint cartilage, a key proliferation zone appears to maintain the elasticity of the surface [48]. The components of the ECM of cartilage, such as proteoglycans, aggrecan, glycosaminoglycans, and hyaluronic acid are key in water fixation. In the deep layers of joint cartilage, calcifications appear in the ECM, which connect articular cartilage and subchondral bone [49]. In this region, chondrocytes undergo apoptosis and the tissue serves as a template for trabecular bone formation. If any of these mentioned components are disturbed, cartilage homeostasis can be severely affected [50,51].

In pathological conditions, such as inflammation, oxidative stress, or increased mechanical strength, the structure of cartilage ECM can be disintegrated and lead to cartilage degeneration [52]. Therefore, an adequate internal structure of the joint cartilage is key for optimal load capacity and function. In this context, the knee joint is one of the parts of the body that is most affected by overuse, such as in the case of cyclists [53,54].

Epidemiological studies carried out on cyclists have shown that 14.8–33% of the population analyzed experience knee pain or injury [55,56,57]. Among the factors causing this type of non-traumatic injury, such as knee OA, is the height of the saddle. Thus, cyclists pedaling on a low saddle height could modify the ankle and/or hip angles (through changes in activation and muscle strength), which could minimize the load transferred through the knee joint [58].

In cycling, hips and knees are the joints that perform greater ranges of motion in a pedaling action. The knee experiences approximately 75° of flexion on average, ranging from 110° of flexion when the crank arm is in the upper position of pedal motion to 25–35° of flexion in the lower position [59]. During the power phase when the pedal goes down, both the hip and knee joints extend in the sagittal plane, while ankle movement is more variable. This usually goes from dorsiflexion in the first half of the power phase to plantar flexion at the end of the descending phase. The force applied to the crank arm, which gives rise to the forward movement of the bicycle, occurs at 90° (crank arm in horizontal position) [59,60].

Altogether, knee overuse injuries are one of the most common problems affecting professional and competition cyclists riding around 20,000 km/year [57,61]. However, in studies conducted on recreational cyclists (non-competitive), knee injuries have also been found to be prevalent, affecting between 24% and 62% of the participants studied [62,63].

## 5. Key Aspects of Knee Osteoarthritis in Cycling Compared to Other Sport Disciplines

Notwithstanding being a frequent pathology in cycling, this is the first review that attempts to propose some pathophysiological mechanisms that would act during the inflammatory process of knee OA in this sport discipline. To this end, a search in the main scientific databases was conducted: Medline/Pub-Med, Web of Science (WOS) and SCOPUS. The search used a combination of different keywords. The most general terms (inflammation AND osteoarthritis AND sport) gave 200 candidate publications. The introduction of two additional keywords (knee AND cyclist) showed in five publications (Table 1).

The few published studies on knee OA in cyclists (Table 1) have not addressed inflammatory aspects in detail. Therefore, this section will try to provide an explanation from a comparative point of view with other sports disciplines where impact actions occur.

OA usually appears as a result of mechanical overload of the joint, regardless of the type of exercise performed [8]. However, from an etiological point of view, patients differ in the underlying cause of the injuries (e.g., traumatic, overload, metabolic, and degenerative) and in the duration, which likely influences the progression of the pathology [68]. Thus, the forces acting on the knee joint can have a high overall impact on the joint surface, on the torsion load, or the overuse of the joint. In this context, sports such as football, hockey, among others, involve a great impact or torsion load on the knee joint. However, in other sports such as cross-country skiing or cycling, overuse rather than an impact or torsional load is responsible for knee injuries [14,15].

From a histopathological point of view, early OA affects both articular cartilage and subchondral bone. In addition, alteration of the pericellular matrix of articular chondrocytes is observed, linked to abnormal activation of cell surface receptors [69]. In this context, the synthesis of surface proteins (e.g., lubricin) is altered, implying a deterioration of surface lubrication and an increase of friction [24,25,26]. This fact could explain the increased inflammation in the knee, which in the cyclist is key due to the important overuse of the joint. In this context, a professional cyclist travels approximately 25,000 km/year (equivalent to 80 pedals/min) including training and competition. This requires a great muscular demand, requiring stabilizing structures for the correct biomechanics of the knee.

In this context, cycling-related muscle damage (concentric movements) can be compared to eccentric sport disciplines. Therefore, in a previous study conducted by our group, three sports disciplines were studied in which cycling (concentric contraction) was compared to two dominant eccentric disciplines (volleyball and basketball). The study indicated that the circulating levels of muscle markers derived from exercise-induced muscle damage were higher in basketball and volleyball players during a training period. However, exercise-induced muscle damage during competition was significantly higher in cyclists due to increased muscle overuse [2]. In addition, it appears that muscle damage may correlate with the different intensities and duration of actions performed during training and competition. However, from the muscle point of view, both eccentric and concentric exercises produce a disruption of the sarcolemma, as the result of an increased proteolytic activity and inflammatory response, manifested by plasma increases in muscle markers [70,71].

Higher intensity and longer workload duration correlate with higher levels of circulating cortisol, although duration appears to be the most determining variable [5]. In this context, serum cortisol levels are indicative of accumulated stress during sports competition [72,73,74]. Thus, our hypothesis is that the observed changes in cortisol (indicator of stress level) may also reflect the inflammatory effect, in other words, higher inflammation causes greater stress.

Thus, the knee joint is prone to damage due to mechanical stresses during the execution of high-intensity daily exercise. Therefore, chronic joint injuries caused by overwork and trauma are the main factors influencing knee degeneration and the major cause of OA in athletes [75]. Different studies indicate that after the onset of inflammation, blood levels of IL-1β, IL-6 and TNF-α increase [75,76,77,78,79]. These increases are even more evident in patients diagnosed with arthritis caused by injury to knee articular cartilage [80]. In addition, patients with OA display an altered immune function and elevated secretion of cytokines, which could favor damage, affecting cartilage function and metabolism [81]. In summary, the aggravation of the gradual decomposition of the cartilage matrix can lead to irreversible injury to the joint structure in patients, displaying the corresponding clinical symptoms and accelerating the progress of joint injury [82].

## 6. Conclusions

Knee OA in cyclists displays an increased inflammatory response with the release of proinflammatory cytokines, mainly due to the overuse of the knee during pedaling. Considering all these aspects, novel approaches in the prevention and possible treatment of knee articular cartilage injuries in cycling are necessary to implement existing protocols.

## Figures and Tables

**Table 1:** Studies regarding knee osteoarthritis in cyclists.

Ref	Case Description	Treatment	Outcomes	Conclusion
[64]	Male cyclist (62 years old), 12 months after following simultaneous bilateral total knee arthroplasty, presenting impairments in left quadriceps strength (26% weaker than the right) and volitional muscle activation. The left quadriceps displayed a central activation ratio of 0.83, 13% lower than the right quadriceps.	Neuromuscular electrical stimulation was implemented for 6 weeks (16 sessions) to the left quadriceps with intense volitional weight-training program focused on unilateral lower extremity exercises.	25% improvement in left quadriceps femoris’ maximal volitional force. After 18-month follow-up, left quadriceps strength was 4% lower than right quadriceps strength and 6% after 24-month follow-up. Central activation ratio of 0.97 after treatment.	Neuromuscular electrical stimulation and an intense strengthening program of the affected extremity can reverse persistent impairments following total knee arthroplasty.
[6]	Professional cyclist (29 years old and BMI = 20.3 kg/m^2^) with grade II knee osteoarthritis (according to magnetic resonance test). Training several hours/day on the bicycle and running 2 h/week on a treadmill, strengthening upper and lower training muscles. Progressive pain in the joint area of the right knee, pain in lower and middle back and increased general fatigue. Complementary tests: (A) Magnetic resonance indicates narrowing of the joint space and mild bone sclerosis with slight joint effusion. (B) Biomechanical test indicates negative impact due to the height of the seat post, distance from the handlebars to the saddle, and position of the cleats. (C) Power loss test indicates 4% loss of power in the left leg and 5% loss in the right leg. No significant improvement in previous treatment with cryotherapy and NSAIDs.	Treatment of 20 sessions in 2 phases: First phase (8 sessions, 5 days a week): (A) Electro-stimulation (20 min, 150 ms pulse and 150 Hz frequency). (B) Ultrasounds (9 min, 1 W/cm^2^). (C) Passive stretching (15 s) of quadriceps, hamstrings, and adductors (3 series of 10 repetitions with 15 s stretching). (D) Cryotherapy (10 min) with ice pack to the knee area. (E) Kinesio Taping (1 day) to reduce pain. Second phase (12 sessions, alternating days a week): (A) Ultrasounds (same as 1st phase). (B) Kotz electrotherapy for quadriceps and hamstrings (2 × 20 min with 5 min break, 2500 Hz/50 Hz). (C) Passive stretching (same as in 1st phase). (D) Cryotherapy (same as in the 1st phase). (E) Kinesio Taping (same as in 1st phase). Adjustments made on the bicycle: Reducing the height of the saddle (9 mm) and the distance from the handlebars (12 mm) to reduce the tension in the lumbar spine area and in the back of the lower limb area. Modification of the cleats: right (3 mm delay and 4° rotation), left (2 mm delay and 7° rotation).	Less pain and more activity according to VAS and IPAQ scales, respectively. WOMAC test indicates less pain and stiffness, maintaining functional capacity. Power loss test: 2% on the left and 3% on the right. The modification of the delay in the cleats allowed the feet in the ascent phase and descent of the pedal go flatter. In addition, the transmission of force is more uniform. Altogether, pedaling is more effective with lower power loss.	The physiotherapy treatment in the injured knee and bicycle modification to change position and biomechanics has improved patient’s symptomatology.
[65]	Male cyclist (16 years old) with left knee injury after a high-velocity fall while cycling. The anteromedial side of the left knee hit the ground in flexion. After the fall, patient indicated excruciating pain, being unable to bear weight. Examination revealed severe joint effusion, tenderness on posterior, and lateral side of the left knee, no vascular injury, and no neurological deficit. Radiographic examination indicated posterior cruciate ligament avulsion fracture and Segond fracture of lateral tibial plateau fragment.	Surgical intervention: The avulsed posterior cruciate ligament fragment and the lateral tibial plateau fragment were fixed with 3.5 mm one-third threaded cannulated cortical screw and augmented with washer.	Hinged knee brace was applied after surgery. Follow-up indicated no pain on weightbearing position with 90% in Osteoarthritis Outcome Score.	Adequate fixation of both fractures can restore knee stability to prevent early joint degeneration.
[66]	Recreational cyclists (n = 14) (age: 57.1 ± 6.37 years) performing 2-min bouts (80 and 120 W) of cycling at three saddle heights of 40°, 30°, and 20° knee extension angle at bottom crank position. Three-dimensional kinematic, kinetic, and electromyography analyses were performed.	Saddle heights result in no changes in internal knee abduction moment. Increases in saddle height from 40° to both 30° and 20°, reduced the knee extension moment.	Increases in work rate increased both knee abduction and extension moments.	Increased knee extension moment with decreased saddle height is likely to indicate increased knee joint load.
[67]	Male cyclist (30 years old) with total left knee displacement due to a motorcycle accident. One year after reconstructive surgery, the subject started cycling training, participating in road cycling and time-trial events. Over the years, left knee developed osteoarthritis limiting high-intensity training loads.	Low-intensity aerobic training combined with blood flow restriction (LI + BFR-80% occlusion) training protocol: (A) Familiarization (1 session): anthropometry, MVIC, TT20 km test. (B) Control period to monitor cyclist performance (4 weeks): TT20 km test and cycling training. (C) Pre-intervention tests (1 each day): TT20 km test, MIT (VO_2_ peak), TTE, MVIC and CSA-VL (both legs). (D) LI + BFR: 3 weeks, 2 sessions/week, 12 min/session. (E) MIT for power output adjustment. (F) LI + BFR: 3 weeks, 2 sessions/week, 18 min/session. (G) MIT for power output adjustment. (H) LI + BFR: 3 weeks, 2 sessions/week, 24 min/session. (I) Post-intervention tests (one each day): CSA-VL, TT20 km, MIT, TTE, MVIC.	The left leg had a greater increase in MVIC with a discrete increase in the right leg, while both legs achieved similar increases in CSA-VL comparing pre- to post-intervention. Reduced time to complete the TT20 km.	LI + BFR improves TT20 km, aerobic capacity, maximal strength, and CSA-VL.

Abbreviations used: BMI, body mass index; CSA-VL, cross-sectional area of vastus lateralis; IPAQ, international physical activity questionnaire; MIT, maximal incremental test; MVIC, maximal voluntary isometric contraction; NSAIDs, nonsteroidal anti-inflammatory drugs; Ref, Reference; TT20 km, 20 km time-trial test; TTE, time to exhaustion test; VAS, visual analog scale; WOMAC, Western Ontario and McMaster Universities Osteoarthritis Index.

## Data Availability

Not applicable.
